# Molecular Regulation of Antioxidant Defense and Metabolic Reprogramming in Xiaozhan Rice Genotypes: Differential Roles of Salicylic Acid and Melatonin Under Salt Stress

**DOI:** 10.3390/cimb47060432

**Published:** 2025-06-07

**Authors:** Yang Wu, Yongbo Duan, Xifan Luo, Mingjun Li, Hengjie Gao, Wei Zhu, Fei Zhao, Jian Liu, Wenzhong Zhang

**Affiliations:** 1 Tianjin Key Laboratory Intelligent Breeding Major Crops, College of Agronomy & Resources and Environment, Tianjin Agricultural University, Tianjin 300392, China; wuyang991103@163.com (Y.W.); luoxifan9902@163.com (X.L.); mingjunli0425@163.com (M.L.); hengjie.gao@foxmail.com (H.G.); 13920357546@126.com (F.Z.); 2 Anhui Provincial Engineering Laboratory for Efficient Utilization of Featured Resource Plants, College of Life Sciences, Huaibei Normal University, Huaibei 235026, China; yboduan@163.com; 3 Key Laboratory of Hybrid Japonica Rice Genetic Breeding, Ministry of Agriculture, National Hybrid Rice Engineering and Technology Research Centre, Tianjin Sub-Centre, Tianjin 300350, China; zwzswzw@126.com; 4 Rice Research Institute, Shenyang Agricultural University, Shenyang 110866, China

**Keywords:** Xiaozhan rice, salt stress, salicylic acid, melatonin, genotype interactions

## Abstract

Against the background of increasing global soil salinity, exogenous salicylic acid (SA) and melatonin (MT) have attracted much attention for their potential in regulating plant stress tolerance and have become an important research direction for the development of green and sustainable agriculture. In this study, the alleviating effects of different concentrations of SA (100–900 μM) and MT (100–900 μM) on salt stress (50 mM NaCl) and their physiological mechanisms were systematically investigated using the Tianjin specialty rice, Xiaozhan rice, as the research object. The results showed that salt stress significantly inhibited the germination and seedling growth of the two varieties, in which the salt-sensitive variety Jinchuan No. 1 showed significantly higher decreases in root length, plant height, and biomass (54.7–69.1%) than the salt-tolerant variety Jindao 919 (4.0–28.9%). Exogenous SA and MT were effective in mitigating salt stress injury, but there were genotypic differences in their pathways of action. For the first time in *japonica* rice, the genotype specificity of the SA/MT response was clearly revealed: SA dominated the response of salt-tolerant varieties by enhancing antioxidant defences, whereas MT optimized the overall performance of the salt-sensitive varieties through scavenging of reactive oxygen species, and in addition, it was further determined that SA and MT exhibited optimal mitigating effects on both varieties in the 300–700 μM concentration range, showing the best mitigation effect for both varieties. This finding provides an important theoretical basis and technological paradigm for precision stress tolerance cultivation of saline rice, and the application of appropriate concentrations of SA/MT according to genotype specificity to reduce the dependence on agrochemicals is of practical value in promoting green and sustainable production in saline agriculture.

## 1. Introduction

Climate change has continuously exacerbated soil salinization, leading to a growing global footprint in terms of both extent and severity [1,2]. According to the latest data from the Food and Agriculture Organization (FAO), approximately 415 million hectares of global land area are affected by salinization, constituting 6.2% of the world’s total terrestrial surface. China ranks among the most severely impacted nations, boasting an estimated 99.13 million hectares of saline-alkali soils. These are primarily distributed across three key regions: the arid northwest, the Songnen Plain in Northeast China, and the coastal areas of the Huang-Huai-Hai Plain [3,4]. Additionally, roughly 20% of the world’s arable land and 33% of irrigated agricultural areas face varying intensities of salt stress, a trend that poses a serious threat to global food security [5]. In China, the severity of this issue is exemplified by the Tianjin Binhai New Area and the Jinghai area, where long-term accumulation of salinity (low sea level) directly affects the yield of local crops. Concurrently, it triggers a series of secondary environmental consequences, such as soil structural deterioration and decreased water infiltration capacity [6,7].

As a specialty agricultural product of Tianjin, ‘Xiaozhan rice’ functions not only as a carrier of the living heritage of regional farming culture but also as a model of practising eco-friendly agriculture [8]. As of 2024, the planting area of Xiaozhan rice accounted for over 80% of the arable land in Tianjin. This rice variety has also been gradually extended to the Beijing-Tianjin-Hebei urban agglomeration, emerging as a benchmark for the green transformation of regional agriculture. However, the widespread soil salinity problem in the coastal area of Tianjin has severely restricted the large-scale cultivation of Xiaozhan rice and the increase in its yield. Therefore, there is an urgent demand to develop eco-friendly approaches for managing saline lands [9].

Fortunately, multiple chemicals, reagents, hormones, and growth regulators have been developed to mitigate the detrimental effects of soil salinization on rice [10]. Among them, SA and MT, as endogenous plant metabolites, offer the advantages of natural origin and environmental compatibility. Moreover, their large-scale production cost is only about $150/tonne (purity ≥ 99%), which is significantly lower than that of the traditional chemical modifiers [11,12,13,14]. Notably, these compounds degrade easily in soil and do not pose a bioaccumulation risk, aligning with the global trend towards drug reduction and low-carbon agriculture. Studies have indicated that SA can significantly enhance salt stress tolerance in rice by activating antioxidant enzyme systems (e.g., superoxide dismutase (SOD) and catalase (CAT)) and regulating ion transport proteins [15,16], such as SA is involved in the cold tolerance mechanism of citrus by regulating metabolite accumulation through the *TGA2-P5CS1/ICS1* module [17]. In contrast, MT plays a crucial role in maintaining cellular homeostasis by scavenging reactive oxygen species (ROS) and promoting the synthesis of osmoregulatory substances (e.g., soluble sugars and proline) [18,19,20], as in the case of sweetpotato, MT upregulated expression of sodium hydrogen exchanger 2 (*NHX2*), K^+^ transporter 1 (*AKT1*), cation/H^+^ exchanger (*CHX*), CBL-interacting protein kinase 1 (*CIPK1*) and antioxidant enzyme genes are expressed to enhance the adaptability to salt stress [21]. However, existing conclusions are mainly based on studies on other crops or specific rice genotypes (*Nipponbare*), among other things, SA regulates the expression of antioxidant genes through npr1-dependent/non-dependent pathways (e.g., SA-activated *MAPK* cascade pathway revealed by Molecular Plant), and MT enhances salt tolerance by regulating ion channels (e.g., *HKT1*) and the ABA signalling pathway [22,23,24]. There are still large gaps in the mechanisms of SA/MT interactions in different rice genotypes (especially local varieties), such as genotypic differences in signalling pathways, specificity of transporter protein expression, and environmental synergistic effects. In summary, in-depth exploration of the SA/MT interaction mechanisms of specialty varieties, such as ‘Xiao Zhan Rice,’ in combination with genotype-specific pathways is needed, thus facilitating the precise application of SA/MT in regional agriculture [25].

Therefore, salt-tolerant and salt-sensitive types were hereby selected from the above four Xiaozhan rice extension varieties for the study. Moreover, the differences in salt stress tolerance were systematically investigated between exogenous SA and MT, and attempts were made to elucidate the intrinsic mechanism of the two compounds to enhance salt tolerance through the effects of the two compounds on seed germination, seedling growth, and physiological metabolism under controlled conditions. Overall, the outcomes of this study are anticipated to provide actionable insights for optimizing the targeted breeding strategy and the precise application of hormones in rice within the salinized agro-ecosystems in Tianjin. Furthermore, these findings will propel the advancement of agro-ecosystems in salinized areas towards a more environmentally friendly and efficient direction.

## 2. Materials and Methods

### 2.1. Experimental Site and Materials

The experiment was conducted from September to December 2024 in a climate-controlled growth chamber at Tianjin Agricultural University (38°96′ N, 117°12′ E). The artificial climate chamber was commissioned to be constructed by Nanjing Lithgow Instrument and Equipment Co., Ltd. (Nanjing, China), and the controlled environmental conditions were as follows: temperature 26 °C, relative humidity 75%, light cycle 12 h/darkness 12 h, LED cold light source, photosynthetic photon flux density of 150 μmol m^−2^ s^−1^, light homogeneity > 90%, three-layer matrix design and edge light decay < 15%. The experimental materials were Jinyuan U99, Tianlongyou 619, Jinchuan No. 1, and Jindao 919, provided by the Xiaozhan Rice Science and Technology Yard (Baodi District, Tianjin, China). Germination boxes (12 cm × 12 cm) and 96-well hydroponic culture boxes (127 mm × 87 mm × 114 mm, aperture diameter 5.0 mm) were purchased from Haimen Yurong Experimental Analysis Instrument Factory (Haimen, China). NaCl was obtained from Yingda Rare Chemical Reagent Factory (Tianjin, China). SA, MT, and Hoagland’s nutrient solution [26] were purchased from Solarbio Science & Technology Co., Ltd. (Beijing, China). All other common chemicals were of analytical grade.

### 2.2. Experimental Design

Intact rice seeds of uniform size were selected for the experiment. The seeds were surface-sterilized with 75% (*v*/*v*) ethanol for 1 min, followed by immersion in 1% (*v*/*v*) NaClO for 15 min, and subsequently rinsed five times with sterile distilled water to eliminate contaminants. Under aseptic conditions on a clean bench maintained at 25 °C, the sterilized seeds were evenly spread and air-dried for subsequent use.

The germination test was conducted according to the standardized method specified in Germination Test (GB/T 3543.4-1995) [27]. Each rice germination box was lined with two layers of filter paper, and 10 mL (containing 50 mmol/L NaCl and exogenous substances, Table 1) of treatment solution was added. Salt concentrations were predetermined in preliminary experiments, while volumes were added to ensure that waterlogging did not occur in the system. Three replicates were prepared with 50 seeds per box without contacting each other. All boxes were incubated in an artificial climate chamber under controlled conditions. During cultivation, the appropriate solution was supplemented as needed to maintain moisture. Germinated seeds were counted and recorded daily during the period, and destructive sampling was carried out on the 14th day after sowing to determine the indices.

To simulate a saline environment, surface-sterilized seeds were soaked in distilled water for 24 h and then transferred to 96-well hydroponic boxes containing half-strength nutrient solution to ensure normal growth until the one-leaf-one-heart stage (96 wells are filled). The solution was then replaced with full-strength nutrient solution containing 50 mM NaCl and exogenous compounds (SA/MT) (Table 1). The nutrient solution was renewed every 3 days to maintain optimal nutrient balance and support healthy rice growth. After 14 days of cultivation, destructive sampling was performed to evaluate target parameters, with three replicates per treatment.

### 2.3. Measurement of Rice Emergence Growth Parameters

During the germination test, seeds were considered germinated when the radicle length reached ≥ 1× seed length and the coleoptile length attained ≥ 1/2 seed length. The number of germinated seeds was recorded daily in the box until termination at 14 DAS. The following parameters were calculated: GP (1), GR (2), GI (3), and VI (4).

(1) $$GP\%=\frac{N_{5}}{N}\times 100\%$$

Germination Potential (*GP*, %) represents the germination percentage at 5 DAS, *N*_5_ is the number of seeds with normal germination (radicle ≥ 1× seed length) at 5 DAS, and *N* is the total number of seeds tested.

(2) $$GR\%=\frac{N_{14}}{N}\times 100\%$$

Germination rate (*GR*, %) represents the cumulative germination percentage at 14 DAS, *N*_14_ is the number of seeds with normal germination (radicle length ≥ 1× seed length and coleoptile length ≥ 1/2 seed length) at 14 DAS, and *N* is the total number of seeds tested.

(3) $$GI=\sum \frac{G_{t}}{D_{t}}$$

*G_t_* denotes the number of seeds germinated at a specific time point (day *t*), while *D_t_* represents the corresponding germination day number associated with *G_t_*.

(4) VI=GI×S

*S* represents the mean seedling height (cm) at the termination of the germination test.

In two different experiments, 10 uniform seedlings were randomly selected from each group for index measurement. After surface moisture was blotted dry with filter paper, Seedling Length (SL) and Seedling Root Length (SRL) were measured using a vernier calliper (0~300 mm, ± 0.02 mm accuracy). Root length was defined as the linear distance from the root-shoot junction to the tip of the primary root, while plant height was measured as the vertical distance from the stem base to the apical meristem. The fresh weight of the whole plant (SFW) was measured using a precision balance (accuracy: 0.001 g). Each sample was then wrapped in aluminum foil and placed in an oven at 105 °C for 30 min to deactivate enzymes. Subsequently, the samples were dried at 80 °C for 72 h until a constant weight was achieved, after which the dry weight (SDW) was recorded.

### 2.4. Measurement of Rice Seedling Growth Parameters in Simulated Real Stresses

Of the replicate treatments in the hydroponics trial, rice leaves were randomly excised using scissors, ensuring that all samples had identical fresh weights (2.00 ± 0.1 g). The collected leaf samples were immediately wrapped in aluminum foil and flash-frozen in a foam box containing liquid nitrogen for subsequent enzyme activity assays. Subsequent measurements, including plant height (PH), root length (RL), fresh weight (FW), dry weight (DW), and stem base diameter (SD), were conducted following the protocol described in Section 2.3.

### 2.5. Measurement of Physiological and Biochemical Indicators

To analyze physiological and biochemical changes in plant tissues, fresh leaf samples were homogenized in liquid nitrogen. The following parameters were then measured using commercial assay kits (Solarbio Science & Technology Co., Ltd., Beijing, China) according to the manufacturer’s instructions: POD activity [28], SOD activity [29], CAT activity [30], MDA content [31], GSH content [32], soluble sugar content [33] and lignin content [34]. All physiological parameters were quantified using three biological replicates with three technical replicates per assay. Mean values were derived from nine data points (three biological × three technical) unless otherwise noted.

### 2.6. Statistical Analysis

Data processing was performed using Microsoft Excel 2019 (Microsoft Corporation, Redmond, WA, USA). Statistical analyses, including one-way analysis of variance (ANOVA) and significance testing (Tukey’s honestly significant difference [HSD] test, *p* < 0.05), were conducted with IBM SPSS Statistics 26 (IBM Corp., Armonk, NY, USA). The salt tolerance of four rice varieties was evaluated using a fuzzy membership function method. The membership function value was calculated as follows Equation (5):

(5) $$\mu \left(X_{j}\right)=\left(X_{j}-X_{\min}\right)/\left(X_{\max}-X_{\min}\right)$$

where *µ*(*X_j_*) represents the membership function value, *X_j_* denotes the measured value of the *j* indicator, and *X*_max_ and *X*_min_ indicate the maximum and minimum values of the *j* indicator. The coefficient of variation *V_j_* for each indicator was determined using Equation (6):

(6) $$V_{j}=\frac{\sqrt{\frac{\sum_{i=1}^{n}{\left(X_{ij}-\bar{X_{j}}\right)}^{2}}{n-1}}}{{\bar{X}}_{j}}$$

where $\bar{X_{j}}$ represents the average value of the *j* indicator across different growth stages, *X_ij_* denotes the value of the affiliation function of indicator *j* for fertility period *i*, and *V_j_* is the standard deviation of the membership function values for the *j* indicator. The weight coefficient *W_j_* for each indicator was calculated as follows Equation (7):

(7) $$W_{j}=\frac{V_{j}}{\sum_{j=1}^{n}V_{j}}$$

The comprehensive evaluation value (*D*) was determined using Equations (8) and (9):

(8) $$D=\sum_{j=1}^{n}\left[\mu \left(X_{j}\times W_{j}\right)\right]$$

(9) $$\Delta D=D_{CK}-D_{NaCl}$$

Higher Δ*D* values indicated weaker salt tolerance of the evaluated varieties, and the two genotypes with the highest and weakest salt tolerance were selected for correlation analysis and cluster visualization using the ‘correlation plot’ and ‘heat map with dendrogram’ applications in Origin 2024 (OriginLab Corporation, Northampton, MA, USA). All data were normalized using Equation (5) and then analyzed by Principal Component Analysis (PCA) using IBM SPSS Statistics 26 in order to reduce the dimensionality of the evaluation indicators. The contribution rates of communalities were calculated and adopted as weight coefficients (*W_j_*) for corresponding indicators. These weighted parameters were subsequently integrated with fuzzy membership function analysis (using only Equation (8), where *W_j_* represents principal component-derived weights) to comprehensively evaluate salt stress mitigation capacities under different exogenous treatments.

## 3. Results

### 3.1. Analysis of Growth Differences and Mechanism in Warble Xiaozhan Rice Under Salt Stress

Salt stress significantly inhibited the growth status (D-value, Supplementary Materials S1) of the experimental rice, with the salt-sensitive cultivar Jinchuan No. 1 exhibiting the largest decline (Δ = 0.242), while the salt-tolerant cultivar Jindao 919 showed the smallest reduction (Δ = 0.122) (Table 2). Comparative analysis revealed these differential responses stemmed from fundamentally distinct physiological regulation strategies: Jindao 919 maintained ionic homeostasis through enhanced antioxidant enzyme activities, whereas Jinchuan No. 1 displayed higher salt sensitivity due to insufficient oxidative damage repair capacity. Notably, the substantial difference in salt tolerance (63.8%) between Tianlongyou 619 (Δ = 0.216) and Jinyuan U99 (Δ = 0.132) further corroborated the critical influence of genetic background on salt stress responses. Based on the comprehensive consideration of significant phenotypic differences and representative mechanisms, Jinchuan No. 1 and Jindao 919 were selected for the comparison of physiological regulation strategies under salt stress.

### 3.2. Effects of Different Exogenous Substances on Seed Germination Characteristics of Rice Under Salt Stress

As shown in Table 3, the germination characteristics of the salt-tolerant variety Jindao 919 and the salt-sensitive variety Jinchuan No. 1 showed significant differentiation under salt stress. The GR and VI of Jindao 919 remained relatively stable under NaCl treatment, while several germination parameters of Jinchuan No. 1 were significantly suppressed, revealing that its salt-sensitive genotypes were more responsive to ionic toxicity at the early germination stage. Interestingly, the vigour index of Jinchuan No. 1 was generally higher than that of Jindao 919 under exogenous treatments, but its stress recovery threshold was significantly lagged, speculating that salt-tolerant varieties may achieve damage repair through rapid mobilization of preexisting metabolites.

The regulatory effect of exogenous SA was dose-specific, with the S3 treatment (500 μM SA) showing a significant gain in both varieties: an 18.6% increase in the germination rate of Jindao 919 and a 33.5% increase in the vigour index of Jinchuan No. 1. In contrast, MT treatment showed a genotype-dependent ‘dose-effect difference’: 500 μM MT increased biomass accumulation of Jinchuan No. 1 by 22.4%. whereas Jindao 919 showed significant gains in response to a gradient of MT concentration. In addition, the response of Jindao 919 to the MT concentration gradient was flat and only showed a weak promotion trend in the M3 treatment, and it was speculated that there might be a compensatory inhibition mechanism of the melatonin signalling pathway in its germination regulatory network.

### 3.3. Effects of Different Exogenous Substances on Seeding Growth of Rice Under Salt Stress

As shown in Table 4, the salt-sensitive variety Jinchuan No. 1 and the salt-tolerant variety Jindao 919 exhibited significant growth differences under 50 mM NaCl stress, but the physiological vulnerability of Jinchuan No. 1 was particularly prominent: its root length, seedling height, Seedling Fresh Weight, and dry weight decreased sharply by 54.7%, 69.1%, 52.4%, and 13.4%, compared with the control, whereas the corresponding reductions in Jindao 919 were only 28.9%, 16.4%, 14.2%, and 4.0%, which was a visual confirmation that the salt-tolerant genotypes are seen to have strong ionic homeostatic and osmoregulatory functions. The differential restorative capacity was particularly pronounced: SA maximized root regeneration in Jinchuan No. 1 (S4, +192.3%), whereas MT optimized photosynthetic partitioning in Jindao 919. This organ-specific efficacy highlights SA’s superiority in root signalling and MT’s role in shoot resource allocation.

The restorative effect of exogenous SA treatment on salt-sensitive varieties was even more significant, with Jindao 919 reaching its peak growth at S3, whereas Jinchuan No. 1 demonstrated a wonderful restorative effect at higher concentrations of S4, with root length increasing by 192.3% to 14.24 cm and dry weight even exceeding that of the control by 8.3%, revealing that SA can break through the salt damage threshold by activating the root regeneration pathway. It is noteworthy that the root development of both varieties responded better to SA than MT treatment, suggesting that SA may occupy a core regulatory node in root-crown signalling. In contrast, the effect of MT showed a typical genotype-concentration interaction pattern: the salt-tolerant variety Jindao 919 showed a 41.6% increase in root elongation and biomass under the M2-M3 gradient, suggesting that the medium concentration of MT optimized its resource capture strategy by enhancing ROS scavenging efficiency, whereas the salt-sensitive variety Jinchuan No. 1 showed a 37.2% increase in plant height and an 18.4% increase in dry weight under the high concentrations of M3-M4, suggesting that the above-ground part of the root crown signalling was enhanced by MT-mediated redistribution of photosynthesis products.

### 3.4. Effects of Different Exogenous Substances on Plant Morphogenesis of Rice Under Salt Stress

Salt stress showed significant genotype-specific physiological inhibition in the two rice varieties (Table 5). In the salt-sensitive variety Jinchuan No. 1, the root length dropped sharply from 14.89 cm to 4.52 cm (a 69.6% decrease) when exposed to 50 mM NaCl, which was a much larger drop compared to the salt-tolerant variety Jindao 919, which only decreased by 54.7% (from 10.29 cm to 4.66 cm). Plant height, fresh weight, and dry weight indicators simultaneously corroborated this trend: the corresponding indicators of Jinchuan No. 1 decreased by 69.1%, 52.4%, and 13.4%, while those of Jindao 919 decreased by only 16.4%, 14.2%, and 4.0%, which highlighted the innate resistance advantage of salt-tolerant genotypes through ionic compartmentalisation and osmoregulation mechanisms.

The exogenous SA treatments showed distinct varietal suitability: The salt-tolerant variety Jindao 919 grew taller and heavier, reaching 16.92 cm and 23.00 mg under the S3 treatment, which was the highest among all treatments. Meanwhile, the salt-sensitive Jinchuan No. 1 returned its dry weight to the normal level of 22.33 mg and increased its stem thickness to 2.89 mm under the same treatment. This indicates that SA greatly improves its ability to adapt by activating certain metabolic processes, and that SA helps root growth more effectively than MT in both varieties, possibly due to its role in regulating signalling. In contrast, MT treatment showed a complicated interaction between concentration and genotype. In contrast, MT treatment showed a complex concentration-genotype interaction pattern. The salt-tolerant variety Jindao 919 synergistically increased plant height (16.63 cm) and stem thickness (3.14 mm) at M3 concentration, while Jinchuan No. 1 plant height soared to 23.10 cm at M2 concentration (e.g., 300 μM MT), which was 28.6% higher than that of other treatment groups. However, MT showed a weak improvement in root length and fresh weight with significant concentration fluctuations, which may be limited by genotype-specific melatonin receptor abundance or membrane transport efficiency.

### 3.5. Regulatory Effects of Different Exogenous Substances on Physiological Metabolism in Salt-Stressed Rice Seedlings

#### 3.5.1. Antioxidant Enzyme System Response

**Figure 1:**
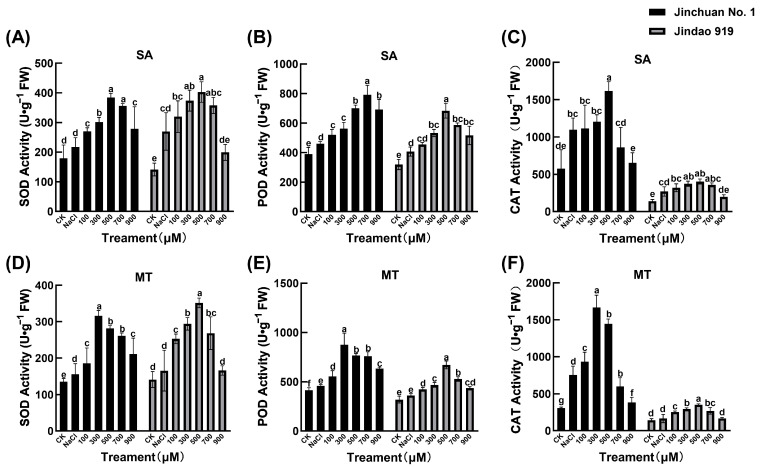
Effects of SA and MT on the SOD (**A**,**D**), POD (**B**,**E**), and CAT (**C**,**F**) activity of rice seedlings under salt stress. Different lowercase letters indicate significant differences at the 0.05 probability level (*p* < 0.05), determined by one-way analysis of variance (ANOVA) and Tukey’s HSD post hoc test for significance. The vertical bar chart represents the mean ± standard deviation (SD) calculated from three repetitions.

Salt stress markedly suppressed antioxidant enzyme activities in both salt-tolerant Jindao 919 and salt-sensitive Jinchuan No. 1, though SA and MT exhibited mechanistically distinct remediation patterns across cultivars (Figure 1). Under salinity, Jindao 919 showed 18.7%, 22.1%, and 15.3% reductions in SOD, Peroxidase (POD), and CAT activities, while Jinchuan No. 1 suffered greater declines (SOD: −26.5%, POD: −31.2%, CAT: −20.8%), confirming heightened vulnerability of stress-sensitive genotypes’ antioxidant systems.

The physiological indices of Jinchuan No. 1 and Jindao 919 exhibited significant differences under SA treatment (Figure 1A–C). Several indices of Jinchuan No. 1 showed a tendency of increasing and then decreasing, suggesting that low concentrations of SA may activate its defence or metabolism pathway, whereas high concentrations of SA triggered the inhibitory effect. In contrast, some indicators of Jindao 919 consistently increased or stayed the same as SA concentration went up, showing a more cautious way of adapting to SA. The differences between the two varieties may stem from the differences in genotypic sensitivity to the SA signalling pathway. In the MT treatment group (Figure 1D–F), Jinchuan No. 1 showed a stronger dynamic response, such as obvious fluctuations in CAT after MT treatment. In contrast, Jindao 919 was relatively stable with small changes in various indexes, such as the CAT level, indicating that its cellular homeostatic mechanism has a high inhibitory capacity against the disturbance of MT, and this difference may be related to the basal activity levels of antioxidant enzyme systems (e.g., SOD, POD) or melatonin receptor expression of the two species. Critically, antioxidant regulation diverged by genotype-hormone interaction: SA preferentially enhanced SOD in Jindao 919 (+76.9%), while MT maximally activated CAT in Jinchuan No. 1 (+37.2%). This indicates SA’s dominance in reinforcing endogenous defences of tolerant varieties, whereas MT compensates for defective ROS scavenging in sensitive lines.

#### 3.5.2. GSH and Soluble Sugar Content Dynamics

**Figure 2:**
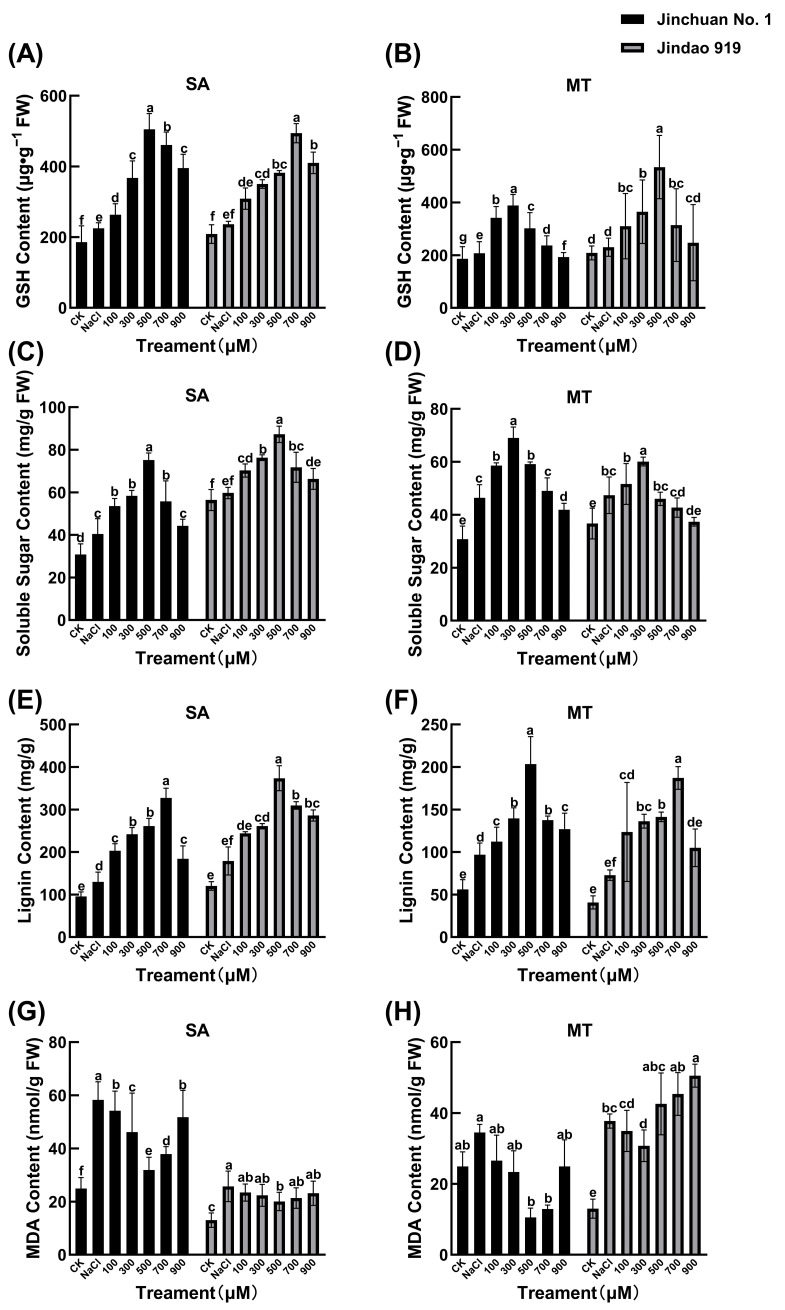
Effects of SA and MT on the GSH (**A**,**B**), soluble sugar (**C**,**D**), lignin (**E**,**F**), and MDA (**G**,**H**) content of rice seedlings under salt stress. Different lowercase letters indicate significant differences at the 0.05 probability level (*p* < 0.05), determined by one-way analysis of variance (ANOVA) and Tukey’s HSD post hoc test for significance. The vertical bar chart represents the mean ± standard deviation (SD) calculated from three repetitions.

Salinity stress-induced metabolic constraints further elucidated the mechanistic divergence of SA and MT interventions across rice cultivars (Figure 2A–D). The salt-tolerant Jindao 919 exhibited a 41.5% reduction in glutathione (GSH) content under stress, whereas the salt-sensitive Jinchuan No. 1 suffered greater depletion (53.2%), confirming compromised antioxidant buffering capacity in stress-vulnerable genotypes.

The exogenous SA significantly enhanced the GSH biosynthesis capacity of the salt-tolerant variety Jindao 919 by targeting the activation of key glutathione synthesis genes and the GSH content of the salt-tolerant variety Jindao 919 was restored to 89.3% of the control level under the treatment of S3, which was much higher than that of the salt-sensitive variety Jinchuan No. 1, which was 45.3%, revealing the molecular advantage of salt-tolerant genotypes to maintain the redox homeostasis through the highly efficient antioxidant system. advantage. Complementarily, MT preferentially alleviated osmotic stress in Jinchuan No. 1 through the soluble sugar synthesis pathway, and its soluble sugar content surged to 104.33 mg g^−1^ (126.8% higher than the control) under M5 treatment, while that of Jindao 919 increased by only 33.4%, suggesting that the sensitive cultivar responded rapidly to osmotic imbalance through reprogramming of sugar metabolism, but the high SA concentration exposed the osmotic stress in Jinchuan No. 1 to a higher level of osmosis. However, the high concentration of SA exposed the metabolic vulnerability of Jinchuan No. 1: its soluble sugar content fell back by 11.1% from the peak, which was more than the 13.5% decrease in Jindao 919, suggesting that the buffering capacity of the sensitive genotypes against exogenous interventions was limited by the insufficient redundancy of metabolic networks.

#### 3.5.3. Lignin Biosynthesis and MDA Accumulation

Salt stress-induced membrane lipid peroxidation (MDA) and lignification processes showed significant genotypic differentiation (Figure 2E–H), with the MDA content of the salt-tolerant variety Jindao 919 elevated by 76.9% under salt stress, whereas the salt-sensitive variety Jinchuan No. 1 showed an increase of 92.4% in MDA.

Exogenous regulatory pathway analysis revealed that MT and SA repaired the damage through differentiated mechanisms. SA preferentially alleviated cell wall lignification in Jindao 919, and the S3 treatment suppressed its lignin content to 1.2-fold of the salt stress level (23.1% reduction), far exceeding the 8.7% improvement in Jinchuan No. 1. The ability of MT to directly scavenge ROS was prominent in Jinchuan No. 1, and the M5 treatment reduced its MDA content by 55.7%, which was significantly better than the 42.3% reduction in Jindao 919. In addition high-dose exogenous treatments exposed the metabolic vulnerability boundary between the two varieties: the S5 concentration triggered a rebound of lignin content in Jinchuan No. 1 to 1.1-fold of the salt-stress level (10.2% increase), whereas in Jindao 919 it only slightly increased by 4.3%; similarly the genotypic differences in the efficacy of ROS scavenging by excess MT (M5) continued to be amplified, with MDA inhibition in Jindao 919 attenuating to 28.5%, whereas Jinchuan No. 1 still maintained a significant improvement of 44.6%, confirming that the redox homeostasis of salt-tolerant varieties is more susceptible to disturbances by dose overload.

### 3.6. Correlation Data and Principal Component Analysis

**Figure 3:**
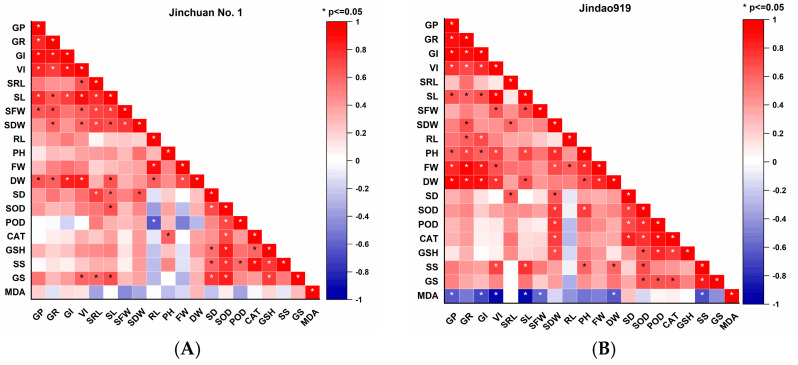

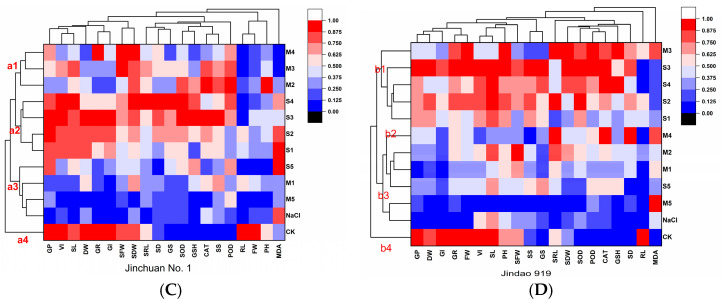
Correlation analysis (**A**,**B**) and cluster analysis (**C**,**D**) of the seedling growth rate, seedling biomass, plant morphogenesis, and physiological indexes of rice seeds. * indicates significant differences at *p* < 0.05 levels. Different red lowercase letters indicate different categories in cluster analysis.

Correlation analysis revealed significant positive correlations (*p* < 0.05) between physiological parameters and exogenous SA/MT treatments in Jinchuan No. 1, indicating effective alleviation of salt stress inhibition through hormonal regulation (Figure 3A). In contrast, Jindao 919 exhibited weaker correlations across most parameters, with only marginal positive associations between certain antioxidant enzymes and MT treatments, suggesting limited salt tolerance enhancement (Figure 3B). Cluster analysis further distinguished the response patterns between cultivars: Jinchuan No. 1′s parameters aggregated into a high-response cluster with strong SA-MT treatment linkage, demonstrating robust synergistic regulation, whereas Jindao 919′s indicators dispersed in low-response clusters, showing blurred treatment boundaries, reflecting inefficient hormonal signalling (Figure 3C,D).

Notably, SA preferentially enhanced osmoregulatory substance accumulation, while MT predominantly strengthened antioxidant defence systems, exhibiting complementary mitigation effects. By comparison, Jindao 919 displayed attenuated responses to both hormones, particularly showing non-significant improvements in osmoregulation. These findings align with the cultivars’ differential salt tolerance capacities, revealing that SA/MT-mediated mitigation efficacy fundamentally depends on genetic predisposition to stress adaptation.

### 3.7. Comprehensive Evaluation Methodology Analysis

PCA systematically elucidated cultivar-specific differences in physiological and morphological regulation pathways underlying salt tolerance in rice varieties (Jindao 919 and Jinchuan No. 1) under exogenous SA and MT treatments (Supplementary Materials S2, Tables S1 and S2). The salt-tolerant Jindao 919 exhibited superior performance, with its first principal component (PC1, 51.37% variance) predominantly governed by high-loading germination parameters (germination potential [GP, 0.850] and germination rate [GR, 0.901]) and antioxidant enzymes (SOD 0.741, POD 0.603, CAT 0.592), demonstrating core salt tolerance mechanisms through synergistic seed vigour and endogenous antioxidant systems. The secondary component (PC2, 21.75%) featured Stem Diameter (SD, 0.669) and glutathione (GSH, 0.650), indicating auxiliary regulation via morphological development and redox homeostasis. In contrast, the salt-sensitive Jinchuan No. 1 showed compromised tolerance, with its PC1 (44.70% variance) dominated by VI (0.896), FW (0.804), and osmolytes (proline, 0.558), suggesting greater reliance on biomass accumulation and osmotic adjustment. PC2 (22.80%) displayed significant loadings for RL (0.875) and MDA (−0.083), highlighting root impairment and membrane peroxidation as key limiting factors under salinity. PCA resolved hormone-genotype specificity: Jindao 919 achieved peak mitigation via SA-activated antioxidant genes, whereas Jinchuan No. 1 relied on MT-driven osmoprotection. This quantifies the core dichotomy: antioxidant reinforcement dominates tolerant varieties, and osmotic adjustment prevails in sensitive lines.

Exogenous treatment showed different regulatory pathways: Jindao 919 exhibited higher SA sensitivity by directly activating antioxidant enzyme genes (such as SOD and CAT), and the comprehensive index reached the maximum value at S3. On the contrary, Jinchuan No.1 has a priority response to MT (optimal M5) and shows enhanced tolerance through the elimination of ROS and proline biosynthesis. Although SA has a certain improvement effect on the RL of Jinchuan No. 1, its effect is still not as good as that of MT. Meanwhile, Jindao 919 shows a limited morphological response to MT and mainly relies on its inherent antioxidant capacity to alleviate salinity.

A comprehensive evaluation resolved that salt stress significantly suppressed the overall performance of both varieties, but there were significant differences in the intensity of their genotypic responses (Table 6). The ranking of the D value of the salt-sensitive variety Jinchuan No. 1 plummeted from seventh to 11th place in the control (CK) under NaCl treatment, while the salt-tolerant variety Jindao 919 was suppressed (ranked from 3rd to 11th place in CK), but the decrease was relatively moderate, confirming the stability of its endogenous salt-tolerance mechanism. The exogenous SA and MT treatments showed a dose-dependent regulation: Jinchuan No. 1 was the top D-value in the M3 treatment, with a synergistic increase in plant height and biomass, while Jindao 919 reached the peak D-value in the S3 treatment (ranked first), with a significant optimization of photosynthetic efficiency and ionic homeostatic indexes. Under the control condition, Jindao 919 showed basic salt tolerance (D value ranked third), and its cellular osmoregulation and antioxidant system preconditioning capacity were the key; whereas, the D value of Jinchuan No. 1 (ranked seventh) declined precipitously under salt stress, revealing the inherent defects of its ion efflux and ROS scavenging mechanism. After salt stress, Jindao 919 maintained ion homeostasis by rapidly up-regulating the expression of salt-sensitive genes, whereas the lag in the activation of the SOS pathway in Jinchuan No. 1 resulted in the accumulation of ion toxicity, and this genotypic difference provides a key phenotypic marker for breeding for salt tolerance.

Among the SA treatments, the S3 treatment produced broad-spectrum gains in both varieties: Jinchuan No. 1 rose to the 2nd place in D value, with increased root vigour and proline content, while Jindao 919 strengthened the cell wall barrier through SA-mediated lignin deposition, with a stable first place in D value. In contrast, MT treatment showed a ‘genotype-dose’ interaction polarity: Jinchuan No. 1 relied on the MT-activated SOD/POD system for ROS scavenging in M3 treatment, while Jindao 919 had a discrete response to MT and even inhibited GSH synthesis at high concentrations (e.g., M5), suggesting that there was a saturating threshold of melatonin signalling in its antioxidant network. SA is superior to MT in terms of cross-species generalization, while MT is more adapted to the emergency repair needs of sensitive genotypes.

## 4. Discussion

Under the ongoing impact of global climate change, soil salinization is deteriorating steadily, presenting a major threat to agricultural productivity and food security. In contrast to previous work focusing on the universal salt tolerance effect of SA or MT [35,36], this study took Xiaozhan rice in the saline-alkaline land of Tianjin Binhai as the research object. For the first time, exogenous SA and MT were applied to the salt-tolerant variety, Jindao 919, and the salt-sensitive variety, Jinchuan No. 1, to systematically reveal the differential responses of these two varieties to SA and MT. This discovery not only offers novel insights into the mechanisms underlying plant salt tolerance but also establishes an important theoretical foundation for the sustainable cultivation of Xiaozhan rice in saline and alkaline environments. It holds immense economic and ecological significance [37].

When delving into the effects of exogenous SA and MT on root and root tip development of different rice varieties during the seedling growth stage, many findings were found to be related to previous studies yet showed differences and innovations, such as SA performed better in restoring root length in the salt-sensitive variety Jinchuan No. 1. By contrast, MT performed better in increasing fresh weight of the salt-tolerant variety Jindao 919 [38,39]. Concurrently, while numerous researchers have studied the mitigation of abiotic stresses in plants by exogenous substances, fewer in-depth studies have been carried out on its specific expression among different varieties [40,41]. However, this group’s observations reveal for the first time that SA enhances salt stress adaptation in sensitive genotypes by activating root developmental regulators, whereas MT attenuates biomass loss in sensitive genotypes by indirectly stimulating the biosynthesis of osmoprotectants. Additionally, through gradient experiments, the optimal mitigation concentration of SA and MT (500 μM) was clarified. Meanwhile, the potential risks of high-concentration treatments (e.g., the soluble sugar content of salt-sensitive varieties decreased by 11.1% at a high concentration of SA) were also revealed. This finding is distinct from previous studies that mainly focused on the effective concentration. It provides a dosage guideline for the safe and efficient field application of exogenous substances [41]. By doing so, it avoids the waste of resources or secondary stress due to blind application. Ultimately, it contributes to achieving the sustainable development objectives of green agriculture [42,43].

Salt stress exhibited significant inhibitory effects on the growth and development of different rice cultivars during the plant morphogenesis stage. Notably, the stress-relieving effects of exogenous SA and MT showed a distinct genotype dependence, breaking through the limitations of the traditional single-hormone or generic regulatory strategies. For example, the SOD activity of the salt-tolerant cultivar Jindao 919 was significantly increased by 76.9% after SA treatment—a phenomenon that is consistent with the mechanism by which SA and MT can alleviate oxidative stress by enhancing the activities of antioxidant enzymes, such as SOD and POD, in *Zea mays*, *barley* and other crops [24,44]. However, a fluctuating rebound effect of lignin and MDA content was observed in the salt-sensitive variety Jinchuan No. 1 at high doses of SA/MT (900 µM), whereas no similar phenomenon was observed in Jindao 919. In-depth analyses showed that Jinchuan No. 1 relied more on the MT-mediated inhibition of membrane lipid peroxidation to enhance salt tolerance (e.g., 55.7% reduction in MDA content) due to a weaker basal antioxidant system [45]. This is similar to the mechanism by which MT maintains membrane stability by inhibiting MDA accumulation in crops such as *cotton* and *maize*, also fits Foyer’s theory of redox homeostasis in plants, in which exogenous MT preferentially maintains the integrity of the membrane system by specifically inhibiting the lipid peroxidation chain reaction when endogenous antioxidant capacity is insufficient [46,47,48]. In addition, Yin Yanling et al. demonstrated that MT can compensate for the defective antioxidant enzyme network of salt-sensitive genotypes by activating relateds [49]. In contrast, the enhancement of antioxidant defence capacity of the salt-tolerant variety Jindao 919 after SA treatment was more efficient, which is highly consistent with the physiological mechanism of ‘SA alleviates stress by enhancing plant osmoregulation’ proposed by Nasrin and Lei [50,51]. In conclusion, SA and MT exhibit complementary functions in mitigating salt stress by differentially regulating hormone homeostasis, the antioxidant system, and ion homeostasis. This not only challenged the traditional ‘universal’ regulatory paradigm but also provided a theoretical cornerstone for precision breeding and hormone-targeted application of genotypic characteristics [35].

This study further revealed the genotype-specific regulation of SA and MT by Principal Component Analysis. For instance, considerable studies confirmed that SA and MT could enhance antioxidant capacity, without quantifying their contribution weights in different genotypes [52,53]. SA dominated the integrated response of salt-tolerant varieties (D-value of 0.640), whereas MT dominated the ROS scavenging capacity of salt-sensitive varieties (D-value of 0.708). This finding forges a theoretical foundation for devising a synergistic multi-hormone regulatory strategy. By comparing the physiological metabolic differences between salt-tolerant and salt-sensitive varieties (e.g., the root regeneration capacity of salt-sensitive varieties was increased by 192.3%), the study echoed the idea of lignin’s involvement in plant mechanical support put forward by Daqiu Zhao [54], which revealed the central role of genotype-hormone interactions in salt domestication. Moreover, herein, the analysis of the affiliation function was conducted, and the results revealed that SA and MT showed synergistic effects in salt-sensitive varieties but antagonistic in salt-tolerant varieties. This endeavour fills the gap in the study of salt tolerance mechanisms in *Japonica* rice by using SA/MT and for plant adversity biology (e.g., SA-mediated lignin deposition).

Given the escalating challenges of global soil salinity, improving crop salt tolerance has become a key priority [55,56]. The stress-relieving effects of exogenous SA and MT provide theoretical support for the advancement of saline precision agriculture management strategies [14,57,58,59]. The strategic application of these exogenous substances can effectively improve the growth performance and yield stability of Xiaozhan rice in saline environments, thereby ensuring food security [60]. In addition, this study provides an innovative method to study the mechanism of plant salt tolerance. Future studies should concentrate on deciphering the molecular regulatory networks of SA and MT during salt acclimation, especially their interactions with other stress signalling pathways [61,62]. While these studies will lay the foundation for breeding superior salt-tolerant crop varieties through targeted genetic manipulation, there are still some limitations warranting further consideration. First, this study focused on the physiological effects of exogenous SA and MT under short-term salt stress, while their mechanisms and effects under long-term salt stress were not explored. Long-term salt exposure may induce more complex physiological and molecular adaptations in plants, and further studies are necessitated to elucidate the long-term responses [63]. Second, the controlled climate chamber conditions used for salt stress simulation in saline soils differ significantly from the actual field environment. Field-specific variables, including soil texture, microbial communities, and climatic factors, may exert substantial impacts on plant growth and salt tolerance [64]. Thus, field validation of the results of SA and MT applications is required.

In conclusion, this study systematically revealed the differential mechanisms of exogenous SA and MT in alleviating salt stress and promoting seedling growth in Xiaozhan rice. These findings not only expand the theoretical framework of plant salt tolerance physiology but also propose novel strategies for addressing the issue of soil salinity in agricultural production. In future research endeavours, the emphasis should be placed on exploring the application potential of these exogenous substances across diverse environmental conditions and crop varieties. By doing so, it will facilitate the advancement of sustainable agricultural practices and contribute to green agriculture and environmental protection.

## 5. Conclusions

In the context of global climate change, the problem of soil salinity is becoming more and more serious, posing a serious threat to agricultural productivity and food security. In this study, we comprehensively investigated the differential mechanisms of exogenous SA and MT on the alleviation of salt stress in Xiaozhan rice seedlings by using a salt-tolerant variety (Jindao 919) and a salt-sensitive variety (Jinchuan No. 1). The results showed that 500 μM SA treatment significantly increased the germination rate, antioxidant enzyme activities (SOD elevated by 76.9%, CAT elevated by 47.1%), and the accumulation of protective substances (GSH increased by 124.7%, and soluble sugars increased by 86.1%) in the salt-sensitive variety, Jinchuan No. 1. Meanwhile, the same concentration of MT showed outstanding protective effects on cell membranes, reducing MDA content by 69.5%. These findings highlight the different but complementary roles of SA and MT in the enhancement of salt tolerance: SA improves plant salt tolerance mainly by enhancing the antioxidant defence system, whereas MT attenuates the damage caused by salt stress in plants mainly by enhancing membrane protection. This study provides practical green solutions for agricultural practices in saline environments, e.g., 500 μM SA soaked seeds or foliar sprays can not only significantly improve salt tolerance in Xiaozhan rice, but also reduce the use of chemical fertilizers and pesticides, which in turn reduces the negative impacts on the environment. Further research on the synergistic effects of salt stress and MT and their integration with molecular breeding methods to develop more salt stress-tolerant rice varieties will contribute to sustainable agricultural development in salinity-affected areas, ensure food security stability in the face of the challenge of increasing soil salinity, and make an important contribution to green agriculture and environmental protection.

## Figures and Tables

**Table 1:** Experimental treatment scheme.

Code	Treatment	Code	Treatment
CK	0 mM NaCl + 0 μM SA/MT	NaCl	50 mM NaCl
S1	50 mM NaCl + 100 μM SA	M1	50 mM NaCl + 100 μM MT
S2	50 mM NaCl + 300 μM SA	M2	50 mM NaCl + 300 μM MT
S3	50 mM NaCl + 500 μM SA	M3	50 mM NaCl + 500 μM MT
S4	50 mM NaCl + 700 μM SA	M4	50 mM NaCl + 700 μM MT
S5	50 mM NaCl + 900 μM SA	M5	50 mM NaCl + 900 μM MT

**Table 2:** Comparison of antioxidant enzyme activities and D values of different rice varieties under salt stress.

Cultivar	Treatment	SOD Activity (U g^−1^ FW)	POD Activity (U g^−1^ FW)	CAT Activity (U g^−1^ min)	D Value	ΔD
Jinyuan U99	CK	214.125 ± 16.25	215.66 ± 41.90	776.49 ± 237.76	0.609	0.132
	NaCl	272.275 ± 30.40	324.33 ± 47.92	978.725 ± 97.43	0.477	
Tianlongyou 619	CK	250.37 ± 29.66	272.00 ± 35.38	380.465 ± 120.81	0.656	0.216
	NaCl	278.575 ± 39.23	386.33 ± 23.65	567.615 ± 174.79	0.440	
Jindao 919	CK	141.31 ± 21.68	319.33 ± 34.48	624.79 ± 310.25	0.462	0.122
	NaCl	217.655 ± 59.46	383.66 ± 24.60	828.075 ± 180.73	0.340	
Jinchuan No.1	CK	157.50 ± 26.49	402.00 ± 35.38	442.18 ± 132.73	0.552	0.242
	NaCl	186.41 ± 30.05	459.00 ± 14.28	927.53 ± 136.88	0.310	

**Table 3:** Exogenous SA and MT affect rice seed germination under salt stress (%).

Cultivar	Treatment	Germination Potential (GP)	Germination Rate (GR)	Germination Index (GI)	Vigour Index (VI)
Jinchuan No. 1	CK	95.33 ± 1.15 a	98.66 ± 1.15 a	14.88 ± 0.24 a	140.49 ± 2.26 a
	NaCl	79.33 ± 3.05 b	93.33 ± 1.15 b	10.32 ± 0.31 d	63.00 ± 0.88 e
	S1	90.00 ± 4.00 ab	96.66 ± 1.15 ab	13.39 ± 0.12 b	121.08 ± 1.13 b
	S2	95.33 ± 1.15 a	98.00 ± 2.00 a	14.09 ± 0.69 ab	128.34 ± 6.28 ab
	S3	96.66 ± 1.15 a	99.33 ± 1.15 a	15.02 ± 0.28 a	143.16 ± 2.73 a
	S4	93.33 ± 3.05 a	96.66 ± 1.15 ab	12.96 ± 0.22 c	139.85 ± 2.44 a
	S5	90.00 ± 4.00 a	94.66 ± 1.15 b	11.72 ± 0.27 cd	82.56 ± 1.93 d
	M1	82.66 ± 3.05 bc	94.66 ± 1.15 b	10.62 ± 0.06 d	68.29 ± 0.24 e
	M2	85.33 ± 5.03 bc	94.66 ± 1.15 b	10.94 ± 0.25 d	82.47 ± 1.95 d
	M3	89.33 ± 3.05 ab	95.33 ± 2.30 ab	11.50 ± 0.22 cd	109.25 ± 2.14 c
	M4	93.33 ± 1.15 a	98.66 ± 1.15 a	12.11 ± 0.31 c	77.14 ± 1.98 de
	M5	86.00 ± 2.00 bc	94.00 ± 0.00 b	10.70 ± 0.31 d	47.45 ± 1.07 f
Jindao 919	CK	97.33 ± 1.15 a	99.33 ± 1.15 a	12.87 ± 0.28 a	109.41 ± 2.43 a
	NaCl	72.00 ± 3.46 d	90.66 ± 1.15 e	9.58 ± 0.19 j	73.94 ± 1.67 de
	S1	90.66 ± 3.05 ab	96.00 ± 2.00 abcd	11.08 ± 0.54 cd	86.56 ± 4.29 c
	S2	91.33 ± 4.16 ab	98.00 ± 2.00 ab	11.45 ± 0.39 bc	97.62 ± 3.40 b
	S3	98.00 ± 2.00 a	99.33 ± 1.15 a	12.08 ± 0.18 ab	110.17 ± 1.73 s
	S4	90.00 ± 4.00 ab	96.66 ± 1.15 abcd	10.95 ± 0.28 cde	95.26 ± 2.43 b
	S5	79.33 ± 4.16 cd	94.00 ± 2.00 cd	10.21 ± 0.20 gh	71.01 ± 1.43 e
	M1	74.00 ± 2.00 d	93.33 ± 1.15 d	9.95 ± 0.06 hi	55.75 ± 0.37 g
	M2	80.00 ± 2.00 cd	96.00 ± 0.00 abcd	10.27 ± 0.03 fgh	79.12 ± 0.23 d
	M3	83.33 ± 3.05 bc	97.33 ± 2.30 abc	10.68 ± 0.25 def	62.67 ± 1.46 f
	M4	84.66 ± 1.15 bc	95.33 ± 1.15 bcd	10.38 ± 0.23 efg	51.25 ± 1.16 h
	M5	76.00 ± 2.00 d	92.66 ± 3.05 d	9.78 ± 0.19 i	32.60 ± 0.66 i

The data in the table are the mean ± standard deviation, *n* = 3. Different lowercase letters in the same column indicate significant differences at *p* < 0.05; the same letter indicates no significant difference (*p* > 0.05).

**Table 4:** Effects of SA and MT on growth in rice seedlings under salt stress.

Cultivar	Treatment	Seedling Root Length (SRL, cm)	Seedling Length (SL, cm)	Seedling Fresh Weight (SFW, mg)	Seedling Dry Weight (SDW, mg)
Jinchuan No. 1	CK	10.38 ± 1.53 bc	8.94 ± 0.93 bc	96.65 ± 8.70 ab	19.63 ± 0.73 bc
	NaCl	4.70 ± 1.75 e	2.76 ± 0.15 f	46.00 ± 4.58 e	17.00 ± 1.00 de
	S1	8.57 ± 1.15 cd	9.04 ± 0.57 bc	74.80 ± 6.62 cd	18.43 ± 0.57 cd
	S2	8.93 ± 1.40 cd	9.11 ± 0.54 bc	79.21 ± 6.75 bcd	19.06 ± 0.38 bcd
	S3	10.31 ± 1.36 bc	9.53 ± 0.88 ab	84.01 ± 8.51 abc	19.56 ± 1.50 bc
	S4	14.24 ± 1.17 a	10.79 ± 0.73 a	87.53 ± 11.76 ab	21.02 ± 0.18 a
	S5	7.38 ± 0.80 d	7.04 ± 0.91 de	74.68 ± 10.22 cd	17.46 ± 0.87 de
	M1	8.03 ± 1.61 cd	4.13 ± 0.96 ef	66.66 ± 13.79 de	19.00 ± 2.64 bcd
	M2	10.13 ± 1.72 bc	7.53 ± 1.84 cde	69.00 ± 7.21 de	19.66 ± 2.08 abc
	M3	11.76 ± 1.78 ab	9.50 ± 0.75 ab	104.33 ± 12.50 a	20.00 ± 1.00 ab
	M4	9.13 ± 2.28 cd	6.36 ± 0.32 de	98.33 ± 15.04 ab	20.66 ± 1.52 a
	M5	7.70 ± 2.10 d	3.43 ± 0.51 ef	55.66 ± 11.93 e	15.00 ± 4.00 e
Jindao 919	CK	8.82 ± 1.19 bcd	8.50 ± 0.92 abc	101.08 ± 8.53 abc	19.03 ± 0.29 bcd
	NaCl	6.27 ± 1.37 e	7.11 ± 0.56 de	86.71 ± 3.78 cd	18.28 ± 0.15 de
	S1	7.28 ± 1.11 de	7.81 ± 0.45 bcd	87.05 ± 5.26 cd	18.60 ± 0.28 cde
	S2	10.47 ± 1.85 ab	8.52 ± 0.83 abc	100.71 ± 10.84 abc	19.31 ± 1.06 abcd
	S3	8.44 ± 1.51 cd	9.12 ± 0.67 a	105.05 ± 8.21 ab	21.32 ± 0.46 a
	S4	8.30 ± 0.77 cd	8.70 ± 0.67 ab	101.58 ± 13.34 abc	20.44 ± 0.28 ab
	S5	8.03 ± 0.60 d	6.95 ± 0.64 def	90.88 ± 7.6 b bc	18.26 ± 1.41 de
	M1	8.90 ± 0.60 bcd	5.60 ± 0.45 fg	102.66 ± 11.50 abc	18.66 ± 2.08 cde
	M2	10.20 ± 0.20 abc	7.70 ± 0.78 cd	115.66 ± 11.59 a	20.00 ± 3.60 abc
	M3	10.93 ± 0.80 a	5.86 ± 0.77 ef	88.33 ± 8.38 cd	21.33 ± 3.05 a
	M4	10.43 ± 0.66 ab	4.93 ± 0.41 g	84.00 ± 8.18 d	19.33 ± 5.85 abcd
	M5	6.23 ± 1.23 e	3.33 ± 0.92 h	73.00 ± 13.22 e	17.66 ± 2.51 e

The data in the table are the mean ± standard deviation, *n* = 3. Different lowercase letters in the same column indicate significant differences at *p* < 0.05; the same letter indicates no significant difference (*p* > 0.05).

**Table 5:** Effects of SA and MT on plant morphogenesis of rice under salt stress.

Cultivar	Treatment	Root Length (RL, cm)	Plant Height (PH, cm)	Fresh Weight (FW, mg)	Dry Weight (DW, mg)	Stem Diameter (SD, mm)
Jinchuan No. 1	CK	14.89 ± 1.37 a	17.78 ± 0.38 b	218.66 ± 4.61 a	22.00 ± 0.00 a	1.72 ± 0.14 cd
	NaCl	4.52 ± 0.84 cd	13.10 ± 1.76 efg	110.25 ± 37.62 ef	14.66 ± 0.57 e	1.66 ± 0.13 d
	S1	8.36 ± 0.70 b	15.44 ± 1.07 cde	128.00 ± 5.56 cd	19.66 ± 0.57 b	1.92 ± 0.12 bcd
	S2	10.63 ± 0.46 ab	15.69 ± 1.09 cde	135.66 ± 1.52 c	20.33 ± 0.57 ab	2.60 ± 0.20 a
	S3	5.36 ± 0.28 cd	16.97 ± 0.13 bc	141.33 ± 3.21 bc	22.33 ± 0.57 a	2.89 ± 0.18 a
	S4	4.79 ± 0.89 cd	14.61 ± 0.82 def	113.33 ± 4.16 ef	18.00 ± 1.00 bc	3.21 ± 0.34 a
	S5	4.05 ± 0.27 d	12.12 ± 0.65 fg	92.33 ± 24.58 fg	16.00 ± 1.00 cd	1.36 ± 0.13 e
	M1	9.67 ± 1.88 ab	14.23 ± 1.98 def	154.00 ± 19.16 b	17.00 ± 1.00 cd	1.97 ± 0.09 bc
	M2	5.82 ± 0.61 cd	23.10 ± 2.15 a	129.25 ± 26.66 cd	16.66 ± 0.57 cd	2.05 ± 0.21 ab
	M3	5.23 ± 0.97 cd	16.00 ± 1.74 bcd	119.00 ± 9.30 de	15.33 ± 0.57 de	2.51 ± 0.24 a
	M4	4.81 ± 0.66 cd	14.55 ± 2.27 def	111.75 ± 18.57 ef	13.00 ± 1.00 ef	2.48 ± 0.13 a
	M5	4.25 ± 1.02 d	10.86 ± 2.71 g	90.25 ± 20.12 g	11.33 ± 1.15 f	1.78 ± 0.14 cd
Jindao 919	CK	10.29 ± 0.56 a	13.82 ± 0.42 cd	149.66 ± 17.03 a	21.33 ± 2.51 ab	1.85 ± 0.09 f
	NaCl	4.66 ± 0.14 d	11.87 ± 1.20 ef	99.00 ± 16.69 e	14.66 ± 1.52 f	1.85 ± 0.08 f
	S1	7.14 ± 0.46 b	13.70 ± 0.46 cd	122.66 ± 8.14 cd	20.00 ± 1.00 bc	2.54 ± 0.18 de
	S2	7.49 ± 0.54 b	15.13 ± 0.36 b	138.33 ± 5.13 ab	21.00 ± 1.00 ab	3.05 ± 0.22 bc
	S3	4.97 ± 0.70 d	16.92 ± 0.60 a	147.00 ± 7.54 a	23.00 ± 1.00 a	3.44 ± 0.21 ab
	S4	4.73 ± 0.26 d	14.48 ± 0.60 bc	130.00 ± 9.53 bc	18.13 ± 0.80 cd	2.72 ± 0.21 cd
	S5	4.42 ± 0.21 d	11.04 ± 0.64 fg	122.66 ± 4.72 cd	17.66 ± 2.08 de	1.84 ± 0.17 f
	M1	4.83 ± 1.02 d	12.49 ± 1.63 de	113.00 ± 7.48 d	16.33 ± 1.52 e	2.64 ± 0.38 cd
	M2	6.47 ± 0.43 c	12.60 ± 1.59 de	121.00 ± 13.24 cd	17.33 ± 0.57 de	2.69 ± 0.30 cd
	M3	7.37 ± 1.15 b	16.63 ± 0.47 a	147.00 ± 16.79 a	18.00 ± 1.00 cd	3.14 ± 0.82 abc
	M4	4.86 ± 0.37 d	10.48 ± 0.64 g	119.75 ± 15.08 cd	18.66 ± 1.15 cd	3.70 ± 0.21 a
	M5	4.58 ± 0.04 d	7.36 ± 2.26 h	105.25 ± 35.96 de	14.66 ± 0.57 f	2.24 ± 0.11 e

The data in the table are the mean ± standard deviation, *n* = 3. Different lowercase letters in the same column indicate significant differences at *p* < 0.05; the same letter indicates no significant difference (*p* > 0.05).

**Table 6:** Comprehensive index evaluation of exogenous substances’ capacity to alleviate salt stress.

Cultivar	Treatment	Membership Function				D Value	Rank
Jinchuan No. 1	CK	0.636	0.000	0.877	0.366	0.452	7
	NaCl	0.157	0.552	0.013	0.623	0.240	11
	S1	0.565	0.511	0.196	0.131	0.432	8
	S2	0.785	0.620	0.414	0.405	0.611	3
	S3	1.000	0.770	0.102	0.130	0.701	2
	S4	0.733	0.804	0.043	0.521	0.588	4
	S5	0.405	0.661	0.000	0.397	0.376	10
	M1	0.294	0.704	0.422	0.763	0.400	9
	M2	0.535	0.691	0.489	1.000	0.543	5
	M3	0.656	1.000	1.000	0.595	0.708	1
	M4	0.411	0.958	0.781	0.068	0.515	6
	M5	0.000	0.725	0.505	0.000	0.217	12
Jindao 919	CK	0.620	1.000	0.230	0.406	0.571	3
	NaCl	0.003	0.251	0.796	0.651	0.215	11
	S1	0.583	0.423	0.965	0.500	0.518	4
	S2	0.758	0.396	0.908	0.507	0.584	2
	S3	1.000	0.173	0.808	0.618	0.640	1
	S4	0.912	0.076	0.662	0.022	0.504	5
	S5	0.340	0.185	1.000	0.201	0.330	8
	M1	0.292	0.325	0.329	0.761	0.317	9
	M2	0.584	0.000	0.105	1.000	0.371	6
	M3	0.662	0.102	0.010	0.299	0.349	7
	M4	0.500	0.249	0.000	0.000	0.280	10
	M5	0.000	0.196	0.541	0.100	0.117	12

## Data Availability

The data presented in this study are available in the figures and tables provided in the manuscript.

## Supplementary Materials

The following supporting information can be downloaded at: https://www.mdpi.com/article/10.3390/cimb47060432/s1.

## Abbreviations

The following abbreviations are used in this manuscript:

SA	Salicylic acid
MT	Melatonin
GP	Germination potential
GR	Germination rate
GI	Germination Index
VI	Vigour index
SRL	Seedling Root Length
SL	Seedling Length
SFW	Seedling Fresh Weight
SDW	Seedling Dry Weight
RL	Root length
PH	Plant height
FW	Fresh weight
DW	Dry weight
SD	Stem Diameter
SOD	Superoxide dismutase
POD	Peroxidase
CAT	Catalase
GSH	Reduced glutathione
GS	Lignin
SS	Soluble sugar
MDA	Malondialdehyde
ROS	Reactive oxygen species
PCA	Principal Component Analysis
P5CS1	Delta-1-pyrroline-5-carboxylate synthetase 1
TGA1	TGACG sequence-specific binding proteins 1
ICS1	ISOCHORISMATE SYNTHASE1
HKT1	High-affinity K transporter 1
