# Supplementary material for: Molecular Regulation of Antioxidant Defense and Metabolic Reprogramming in Xiaozhan Rice Genotypes: Differential Roles of Salicylic Acid and Melatonin Under Salt Stress
Source: Curr Issues Mol Biol. 2025 Jun 7;47(6):432. doi: 10.3390/cimb47060432 (PMC12191652; doi:10.3390/cimb47060432)
Supplement: Supplementary file 1 [file cimb-47-00432-s001.zip › Supplementary Materials S2.pdf]

Table S1. Principal Component Analysis of Germination and Seedling Growth Indicators in Jinchuan No.1

Indicator	loading factor				Composite Score Factor	Weight
	PC1	PC2	PC3	PC4		
GP	0.761	0.354	0.285	-0.361	0.916	4.94%
GR	0.751	0.423	-0.038	-0.196	0.782	5.58%
GI	0.763	0.523	0.262	-0.091	0.933	5.32%
VI	0.896	0.335	0.194	-0.036	0.954	4.97%
SRL	0.798	-0.111	-0.328	-0.267	0.828	4.58%
SL	0.932	0.079	0.05	-0.138	0.896	5.37%
SFW	0.704	0.208	-0.444	-0.393	0.891	4.29%
SDW	0.789	0.007	-0.421	-0.004	0.8	4.28%
RL	0.209	0.875	-0.084	0.279	0.894	4.45%
PH	0.529	0.024	-0.411	0.62	0.835	5.14%
FW	0.338	0.804	-0.26	0.355	0.954	5.93%
DW	0.717	0.486	0.315	0.364	0.982	4.90%
SD	0.771	-0.33	-0.106	-0.197	0.753	5.60%
SOD	0.752	-0.602	0.209	0.062	0.976	4.99%
POD	0.365	-0.806	-0.312	-0.161	0.907	4.95%
CAT	0.539	-0.447	-0.009	0.643	0.905	5.37%
GSH	0.662	-0.511	0.295	0.185	0.82	5.17%
SS	0.558	-0.613	-0.008	0.466	0.904	5.78%
GS	0.708	-0.439	0.43	-0.258	0.945	2.75%
MDA	-0.06	0.083	0.935	0.115	0.897	4.94%
Characteristic Root	8.940	4.560	2.315	1.957		
Variance Explained Rate	44.70%	22.80%	11.57%	9.78%		
Cumulative %	44.70%	67.50%	79.07%	88.86%		

Table S2. Principal Component Analysis of Germination and Seedling Growth Indicators in Jindao 919

Indicator	loading factor				Composite Score Factor	Weight
	PC1	PC2	PC3	PC4		
GP	0.850	-0.306	0.111	-0.384	0.976	5.29%
GR	0.901	-0.173	0.334	-0.135	0.971	5.23%
GI	0.774	-0.519	0.218	-0.257	0.982	5.59%
VI	0.814	-0.495	-0.244	0.076	0.973	5.48%
SRL	0.518	0.314	0.604	0.318	0.833	4.80%
SL	0.788	-0.402	-0.375	0.212	0.969	5.58%
SFW	0.610	-0.186	-0.098	0.634	0.818	4.27%
SDW	0.796	0.406	0.202	0.197	0.878	5.25%
RL	0.392	-0.636	0.583	0.077	0.905	4.76%
PH	0.885	0.015	0.037	0.227	0.836	4.25%
FW	0.867	-0.142	0.373	-0.078	0.918	4.99%
DW	0.883	-0.252	0.092	-0.312	0.950	5.14%
SD	0.500	0.669	0.285	-0.188	0.814	4.87%
SOD	0.741	0.527	-0.164	0.067	0.858	5.08%
POD	0.603	0.744	-0.052	-0.119	0.934	4.94%
CAT	0.592	0.759	-0.017	-0.109	0.939	4.85%
GSH	0.508	0.650	-0.032	0.250	0.743	4.42%
SS	0.722	0.010	-0.653	-0.007	0.948	4.47%
GS	0.646	0.295	-0.611	-0.295	0.965	5.28%
MDA	-0.636	0.632	0.387	-0.112	0.968	5.45%
Characteristic Root	10.274	4.351	2.336	1.213		
Variance Explained Rate	51.37%	21.75%	11.68%	6.06%		
Cumulative %	51.37%	73.13%	84.81%	90.87%		
